# Case report: Evolution of catatonic mutism and psychotic symptoms in an adolescent with Down syndrome: transition from Down syndrome disintegrative disorder to anti-N-methyl-D-aspartate receptor encephalitis

**DOI:** 10.3389/fneur.2023.1200541

**Published:** 2023-06-09

**Authors:** Yuki Minamisawa, Mutsumi Sato, Yoshiaki Saito, Fumikazu Takeuchi, Hidehito Miyazaki, Mao Odaka, Ayako Yamamoto, Yoshitaka Oyama, Yoshihiro Watanabe, Saoko Takeshita, Yukitoshi Takahashi

**Affiliations:** ^1^Department of Pediatrics, Odawara Municipal Hospital, Odawara, Japan; ^2^Children's Medical Center, Yokohama City University Medical Center, Yokohama, Japan; ^3^Department of Pediatrics, National Rehabilitation Center for Children with Disabilities, Tokyo, Japan; ^4^Department of Psychiatric Medicine, Odawara Municipal Hospital, Yokohama, Japan; ^5^Department of Psychiatry/Psychiatric Center, Yokohama City University Medical Center, Yokohama, Japan; ^6^Department of Pediatrics, NHO Shizuoka Institute of Epilepsy and Neurological Disorders, Shizuoka, Japan

**Keywords:** Down syndrome, Down syndrome disintegrative disorder, anti-NMDA receptor antibody encephalitis, cytokine, catatonia, Alzheimer's disease

## Abstract

During her first year of junior high school, a 12-year-old Japanese girl with Down syndrome experienced dizziness, gait disruption, paroxysmal weakness in her hands, and sluggish speaking. Regular blood tests and a brain MRI revealed no abnormalities, and she was tentatively diagnosed with adjustment disorder. Nine months later, the patient experienced a subacute sickness of chest pain, nausea, sleep problem with night terrors, and delusion of observation. Rapid deterioration then developed with simultaneous fever, akinetic mutism, loss of facial expression, and urine incontinence. These catatonic symptoms improved after a few weeks after admission and treatment with lorazepam, escitalopram, and aripiprazole. After discharge, nonetheless, daytime slumber, empty eyes, paradoxical laughter, and declined verbal communication persisted. Upon confirmation of the cerebrospinal N-methyl-D-aspartate (NMDA) receptor autoantibody, methylprednisolone pulse therapy was tried, but it had little effect. Visual hallucinations and cenesthopathy, as well as suicidal thoughts and delusions of death, have predominated in the following years. Cerebrospinal IL-1ra, IL-5, IL-15, CCL5, G-CSF, PDGFbb, and VFGF were raised in the early stage of initial medical attention with nonspecific complaints, but were less prominent in the later stages of catatonic mutism and psychotic symptoms. We suggest a disease concept of progression from Down syndrome disintegrative disorder to NMDA receptor encephalitis, based on this experience.

## 1. Introduction

Acute or subacute regression in adolescents with Down syndrome (DS) has been increasingly recognized in the last decade and has been designated variously as Down syndrome disintegrative disorder (DSDD), Down syndrome regression disorder, or unexplained regression in Down syndrome (1–5). Although diagnostic criteria have not been established for DSDD, patients with this illness exhibit social disengagement and diminished interpersonal response, cognitive and linguistic loss, decreased independence in daily activities, and mental manifestations such as apathy, depression, stereotypies, hallucination, and catatonia (2, 3). The prevalence of DSDD seems relatively rare, with 30 cases being identified in a large cohort of 6,000 DS subjects (2). This condition differs from early-onset Alzheimer's dementia (AD) in DS, in that (1) DSDD mostly arise in 10–20 years of age, whereas AD typically affects DS subjects in their forties or later, gradually increasing in prevalence up to 50% by age 60 years; (2) onset is rapid in DSDD whereas insidious in AD; and (3) some proportion of DSDD subjects completely recover from the deterioration phase, which is not achieved in AD.

A multifactorial etiology has been proposed for DSDD, including prior psychosocial stressors, neuroinflammation, and incipient AD pathology and/or developmental neurotransmitter dysregulation (2–4). There has been increasing interest in autoimmune pathogenesis, owing to the higher prevalence of treated hypothyroidism and serum antithyroid peroxidase (TPO) antibodies than expected in the total DS community, as well as the effects of immunotherapies on DSDD to some extent (1, 4). This is substantiated by increased cell counts and protein levels in the cerebrospinal fluid (CSF) of some DSDD patients (4). However, most studies on CSF autoantibodies have produced null results, and the role of humoral immunity in this illness remains unknown (4).

Herein, we present a case of DSDD, with three separate phases: the first, nonspecific complaint and declining speech, the second, autistic regression with acute catatonia syndrome, and the third, psychosis with profuse hallucinations. CSF anti-N-methyl-D-aspartate (NMDA) receptor autoantibody was negative in the first and second phases of the cell-based assay but became positive in the third phase. Unexpectedly, some CSF inflammatory cytokines were elevated in the first phase but less prominent in the second and third phases. They included certain molecules whose involvement in the etiology of AD have been assumed. The relevance of CSF results, as well as the diagnostic boundary between DSDD and NMDA receptor encephalitis (NMDARE) in this instance, are discussed.

## 2. Case description

The girl with mosaic DS had a mild intellectual handicap and had been attending a special educational class since the age of 9, but she had no medical complications as a result of trisomy 21. Her premorbid demeanor was upbeat, and she enjoyed dancing and singing. She was somewhat obstinately diligent in academic works, and would cry with stress when she was called on in class but could not answer well to the questions. No autistic traits had been reported. Her intellectual quotient was assessed as 58 on the Wechsler Intelligence Scale for Children, 4th edition, at the age of 11 years.

**Figure 1:**
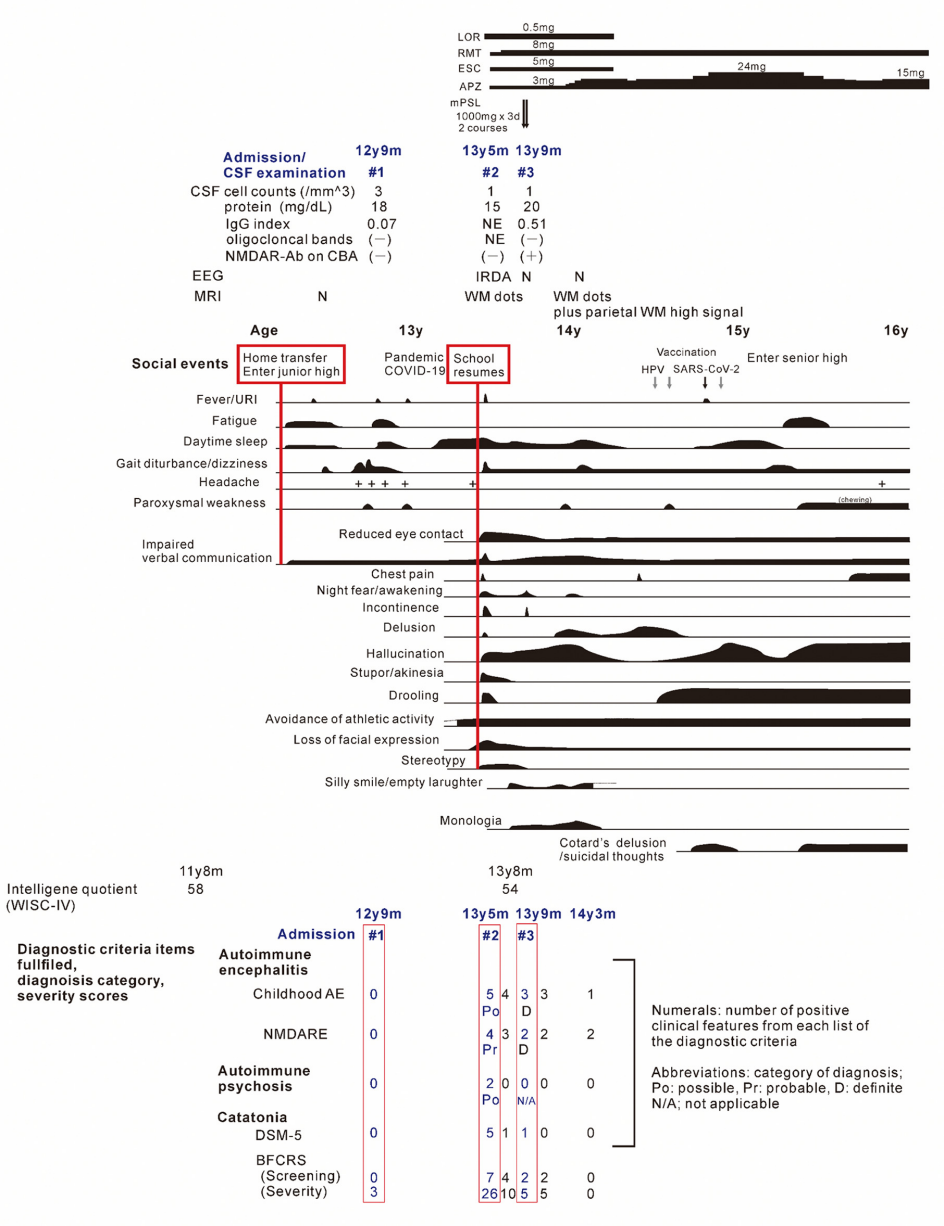
The clinical course of the patient. Periods of hospitalizations are shown at the top of the figure, below the charts of medication. **Bottom**: number of items satisfying the diagnostic criteria and/or scores on diagnostic scales for the childhood autoimmune encephalitis, autoimmune psychosis, and the NMDA receptor encephalitis, and for the catatonia, based on references (7, 8, 12–15). The patient's status was assessed at periods of (1) nonspecific complaints, reduced speech and unsteady gait, (2) on admission due to the catatonia syndrome, (3) on discharge after recovery from catatonic symptoms, (4) before administration of methylprednisolone, (5) after the third discharge, and (6) patient at age 14 years and 3 months. Psychosocial stressors preceding the first and second stage of illness are show in the red-flamed boxes. AE, autoimmune encephalitis; APZ, aripiprazole; BFCRS, Bush-Francis catatonia rating scale; ESC, escitalopram; IRDA, intermittent delta activity; mPSL, methylprednisolone; LOR, lorazepam; N, normal; NMDARE, anti-N-methyl-D-aspartate receptor encephalitis; RMT, ramelteon; WISC-V, Wechsler intelligence scores for children, fifth edition; WM, cerebral white matter.

**Figure 2:**
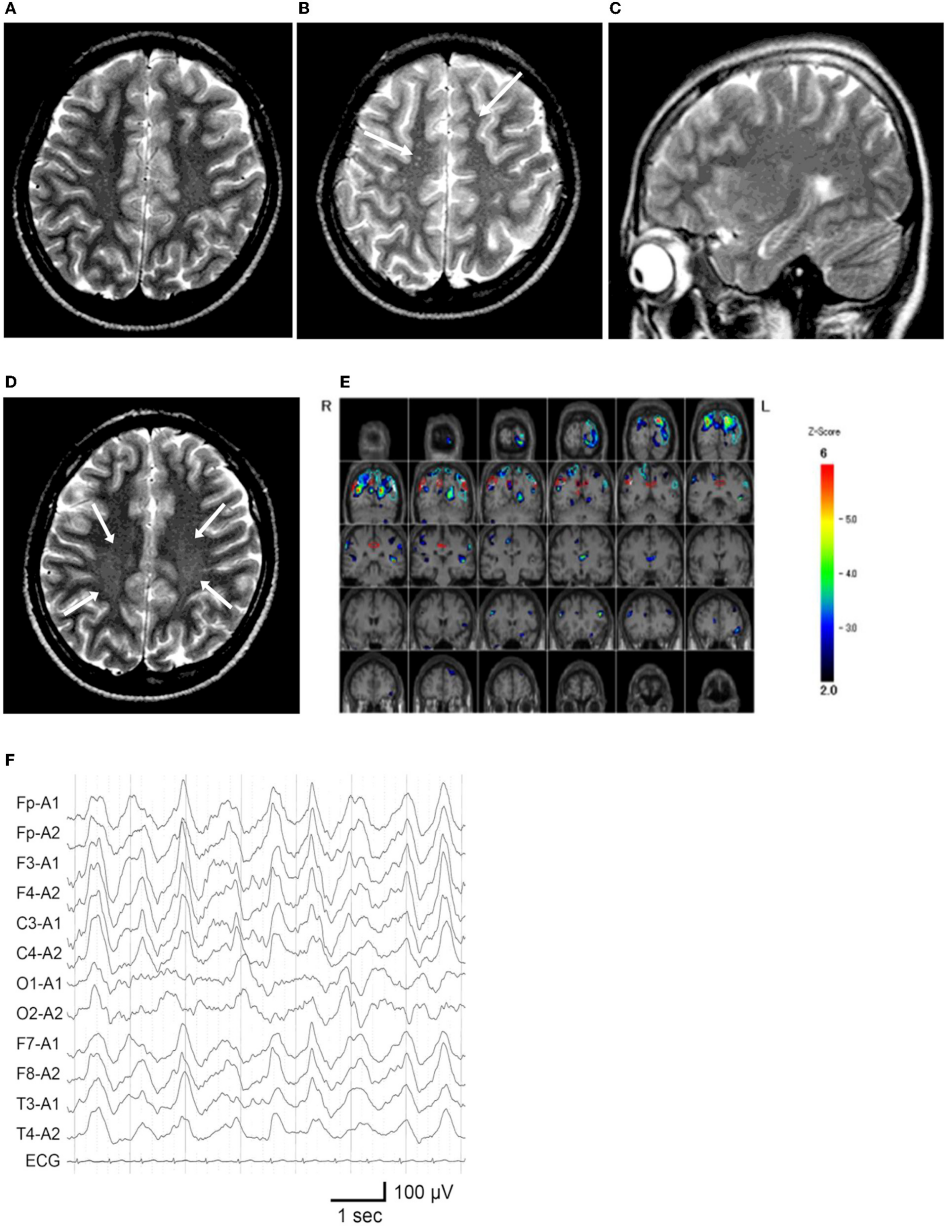
Neuroimaging and electroencephalography of the patient. **(A)** T2-weighted MR imaging at age 12 years and 5 months, during the first hospitalization. **(B, C)** MR images at age 13 years and 5 months, on the second admission owing to catatonic mutism. **(B)** Several high-intensity dots (arrows) are observed. **(C)** The hippocampus appears unremarkable on the para-midline sagittal slice. **(D)** MR image at age 14 years and 1 month, which demonstrated vague high signal areas (arrows) in the bilateral deep parietal white matter. **(E)** ^99m^ Tc-ECD SPECT at age 13 years and 6 months, colored by the easy Z-score imaging system. Hypoperfusion is observed in the bilateral parietal and left occipital areas. **(F)** Electroencephalogram on the first day of admission at 13 years and 5 months. Widespread high-amplitude slow-wave activity is detected.

Shortly after starting junior high school at the age of 12 years, the patient complained of fatiguability and paroxysmal weakening of fingers and acquired an unsteady gait over 3 months period. Slowness in the speech was also observed (Figure 1). Blood tests and brain MRI results were normal (Figure 2A), and the symptoms resolved spontaneously. But, following a summer holiday, the patient had weakness, insomnia, and severe gait disturbance, for which she was taken to the hospital for medical assessments. Brain and spinal cord MRI revealed no specific abnormalities, and routine blood, urine, and CSF samples, analyses, as well as nerve conduction velocity testing, were all unremarkable. She was tentatively diagnosed with adjustment disorder and was discharged. Thereafter nonspecific complaints waxed and waned.

Nine months later, a few weeks after the COVID-19 pandemic was settled down and the school activity restarted, the girl's linguistic communication and eye contact continued to deteriorate, as did her sleep maintenance insomnia, and night terrors. Within a week, she complained of chest pain, nausea, the delusion of observation, and the perception of how each piece of furniture was feeling. The next morning, she was discovered incontinent and unresponsive to her surroundings, her facial appearance being completely lost. When she arrived at our hospital, her body temperature was 38.1°C, which subsided soon after admission. Her awareness level fluctuated substantially, between E4V1M6 and E1V1M4 on the Glasgow Coma Scale. She demonstrated her intent by shaking or nodding her head. No focal neurological signs were noted on examination. However, she was akinetic and completely mute, stared into space, and held a fixed posture or showed waxy flexibility in her extremities. Drooling and retching, were common, as was oral dyskinesia. Blood and CSF tests, including herpes simplex virus DNA, came back within normal ranges. Brain MRI indicated several high-intensity spots in the frontal white matter, but no abnormalities in the hippocampus sections; sleep EEG revealed a widespread high voltage slow-wave activity, that progressed to irregular activity with a frequency range of 3–10 Hz upon awakening (Figures 2B, C, F). Lorazepam was prescribed to treat catatonic syndrome. The patient could talk in sentences the next morning, though as slow as uttering her name in 30 s or more. It was also established that she had experienced visual hallucinations of an unknown person. She could socially smile and laugh a few days later, but she had a prolonged appetite loss, that required tube feeding. The treatment regimen was supplemented with escitalopram, aripiprazole, and ramelteon. On day 14 of admission, she began playing card games, and the stomach tube was withdrawn. She was discharged, with some persistent behavioral and motor issues (Figure 1).

**Figure 3:**
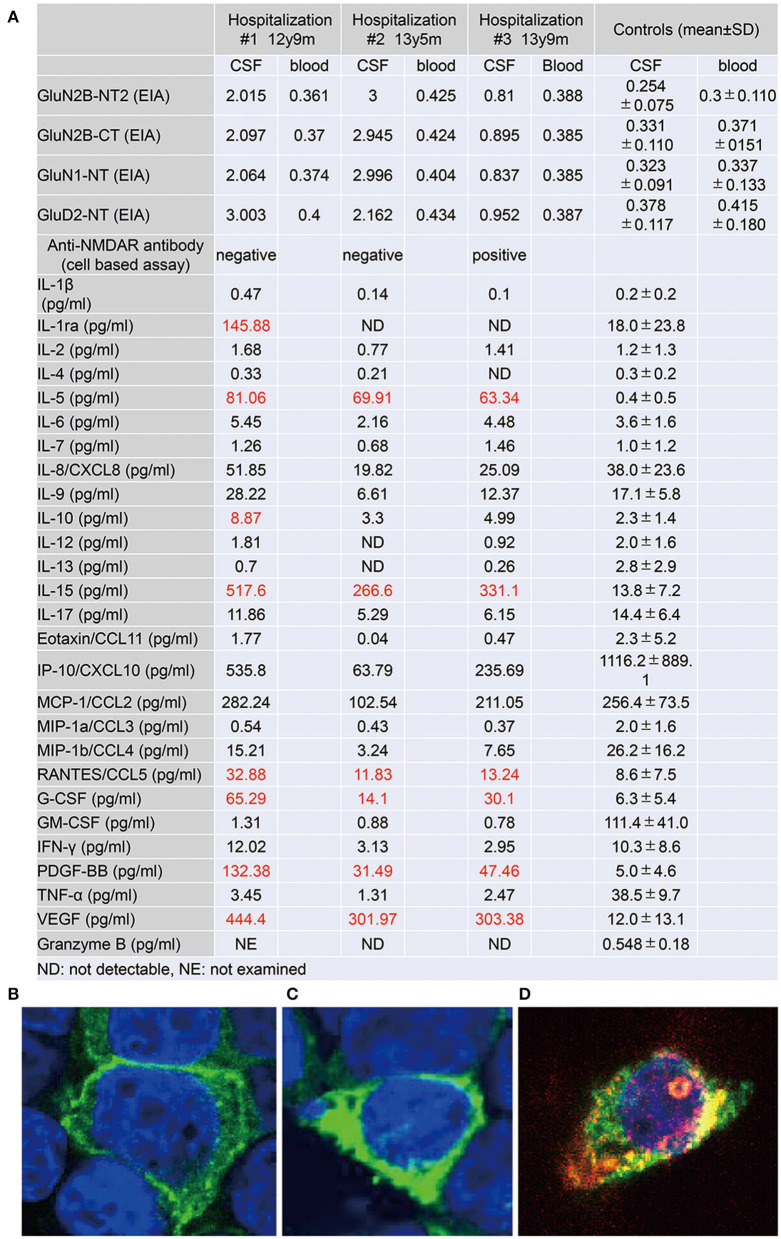
**(A)** Autoantibody to glutamate receptors and levels of cytokines, chemokines, and other factors in the cerebrospinal fluid (CSF) specimens, taken at three distinct phases of illness. Antibodies to GluN2B-NT2, GluN2B-CT, GluN1-NT, and GluD2-NT were measured by ELISA (6). Cytokines, chemokines, and other factors in CSF were measured by multiplex fluorescent immunoassays utilizing the Bio-Plex Pro Human Cytokine 27-plex Assay (Bio-Plex, Hercules, CA) (10). The panel comprises interleukin (IL)-1β, IL-1 receptor antagonist (IL-1ra), IL-2, IL-4, IL-5, IL-6, IL-7, IL-8/CXCL8, IL-9, IL-10, IL-12 (p70), IL-13, IL-15, IL-17, eotaxin/CCL11, fibroblast growth factor-2 (FGF-2), granulocyte colony-stimulating factor (G-CSF), granulocyte macrophage colony-stimulating factor (GM-CSF), interferon-γ (IFN-γ), IFN-γ-induced protein 10 (IP-10)/CXCL10, monocyte chemotactic protein-1 (MCP-1)/CCL2, macrophage inflammatory protein-1α (MIP-1α)/CCL3, MIP-1β/CCL4, platelet-derived growth factor BB (PDGF-BB), regulated on activation, normal T cell expressed and secreted (RANTES)/CCL5, tumor necrosis factor-α (TNF-α), and vascular endothelial growth factor (VEGF). Levels of granzyme B in CSF were measured by the Human Granzyme B ELISA Kit (Cat. No. KT-078, Kamiya Biomedical Company, Seattle, WA, USA) (11). **(B–D)** Results on the cell-based assay (9) using CSF at age 12 y 9 m **(B)**, 13 y 5 m **(C)**, and 13 y 9 m **(D)**. **(B, C)** Cell bodies of cultured HEK cells, transfected with a cDNA coding GluN1/2B, are positively stained (green) by an antibody against the GuN1 epitope but are immunonegative by CSF staining. **(D)** Alongside the immunostaining of GluN1, the cell body is positively stained (red) by the CSF, taken just before the intravenous methylprednisolone therapy. Colocalization (yellow) of the immunostaining by the GluN1 antibody and the CSF is determined.

Soon thereafter, the patient came to manifest with a silly smile, paradoxical laughter, and monology. The patient claimed that there are many, invisible people or goods in her house, that dwarf and fairly are sitting on the air cleaner or her head/arms/clothing, that she was talking with someone in her ear and/or neck, that there are tubes in her ear/mouth/body, and that bad ideas come into her head prompting an urge to stab someone with a pair of scissors. A SPECT examination found hypoperfusion in the bilateral parietal and left occipital lobes (Figure 2E), contradicting the pathophysiology of primary psychiatric illness to explain the catatonia and subsequent psychosis. CSF specimens from both admissions tested positive for anti-NMDA receptor antibodies by enzyme-linked immunosorbent assay (ELISA) (Figure 3) (6). Anti-TPO antibody was found to be positive; but, the patient was euthyroid. Clinical and paraclinical findings in this patient were compatible with DSDD, and at the same time fulfilled the criteria for probable NMDARE (7, 8). However, following points were rather atypical for NMDARE (Figure 1, Supplementary Tables S1, S2); (1) the presence of psychosocial stressors triggering each phase of illness, (2) the first phase of nonspecific complaints as long as 12 months, (3) the order of predominant movement disorder (catatonia) phase and late-evolving psychiatric phase, (4) negative CSF results, and (5) hypoperfusion on SPECT imaging not involving the limbic areas. With a diagnosis of DSDD, intravenous methylprednisolone was provided 3 months after the last admission, at age 13 years and 9 months. However, the effect of this treatment was minimal. Follow-up MRI revealed nonspecific high signal regions (arrows) in the bilateral deep parietal white matter (Figure 2D).

She then claimed to have delusions that her voices were recorded, that another person arose while she was fake, and that the present day was the dinosaur age. These psychotic symptoms were relieved by increasing the dosage of aripiprazole. During the period of preparation for high school admission, however, complaints of somatic symptoms or cenesthopathy predominated including throat discomfort, feeling like her face was slipping off, stubbed or dirty abdomen, dysesthesia in the distal extremities, and difficulty in chewing. Coupled with an auditory hallucination that someone was requesting and/or ordering her to die, the patient was influenced by thoughts over suicide and/or life expectancy, as well as the belief that she was already dead. She is currently a high school student, with persistent psychiatric issues. Such an extended course of hallucinations and delusions prompted us to examine the CSF specimen with cell based assay (CBA), which qualified a positive result of anti-NMDA receptor antibodies for the specimen taken just before the intravenous methylprednisolone (Figure 3) (9). The patient was diagnosed with definite NMDARE, for whom second-line immunotherapies are being planned. Ovarian tumor was not seen on an abdominal MRI.

CSF levels of IL-5, IL-15, CCL-5, granulocyte colony-stimulating factor, platelet-derived growth factor (PDGF)-bb, and vascular endothelial growth factor (VEGF) were raised throughout the course, highest at the first examination at the age of 12 years (Figure 3) (10, 11).

## 3. Discussion

Aside from autoantibody testing, cytokine study on CSF samples from DSDD patients, or even DS people without DSDD, has never been done. This is the first case of DS in which the CSF levels of cytokines and anti-NMDA receptor autoantibodies were measured before, during, and after the subacute catatonic regression. These three times corresponded clinically to the first phase with nonspecific complaints and declining speech, the second period of acute regression and catatonia, and the third phase of psychotic features.

The scores of catatonia symptoms peaked at the second admission (12, 13) (Figure 1), which finalized the syndromic diagnosis of catatonia at this stage. This was fair, however, given the negative CBA results, manifestations at this time also met the clinical symptoms of “possible” autoimmune encephalitis, “possible” autoimmune psychosis, as well as those of “probable” NMDARE (7, 8, 14, 15). Speech deterioration, catatonia, and other cognitive/behavioral difficulties are covered in both categories, indicating overlapping symptoms between DSDD and NMDARE (2, 4, 8). According to the published works (Supplementary Table S1), prior psychosocial stressors often trigger the onset of DSDD, which is not identified in NMDARE. In terms of clinical evolution, NMDARE present in an order of the prodromal phase with symptoms of infection up to 1 week, followed by the psychiatric phase of psychotic, cognitive, and mood disorders lasting weeks to months, and the subsequent neurological phase with movement disorders including catatonia. Auditory and/or visual hallucinations are common in NMDARE during the psychiatric phase, usually preceding catatonia. DSDD is characterized by the acute/subacute regression with depression and/or catatonia. Hallucinations can accompany this regression phase, i.e., not preceding catatonia, in relatively rare occasions. As for the paraclinical data of the patient, lack of pleocytosis, normal protein levels, absence of oligocloncal band, and IgG index in normal range, were not suggestive either of DSDD or NMDARE, some proportion (lower in NMDARE) of patients presenting these unremarkable data in each entity. Serum anti-TPO antibody is rather characteristic in DSDD, but can be detected in rare cases of NMDARE, although its role in the pathogenesis remains unclear in both entities. Extreme delta brush is specific to, but its absence does not reject, NMDARE. Rhythmic delta activity was a nonspecific finding, possibly accompany both. Hyperintensity punctate signals in the cerebral white matter on T2-weighted or fluid-attenuation inversion recovery imaging were nonspecific findings, which can be seen in either NMDARE, DSDD, or even DS without regression. Notably, SPECT hypoperfusion in the parieto-occipital areas were common to DS subjects, whereas hyper-/hypoperfusion was predominant in the limbic systems including the medial temporal, the fronto-orbital, and the cingulate cortex in NMDARE (Supplementary Table S1).

Therefore, clinical features of the present patient until the third admission were typical for DSDD, rather not NMDARE. In the paraclinical data, normal CSF results and SPECT hypoperfusion in posterior areas were unusual for NMDARE. Through these multimodal diagnostics, we assumed that the diagnosis of DSDD was most appropriate for the patient. However, the patient began to experience profuse and extended hallucinations during and after the recovery from the catatonia and autistic regression. This evolution coincided with the positive CBA results of the cell-surface immunolabelling of cultured neurons expressing GluN1/GluN2B, when the criteria for “definite” NMDARE were fulfilled (7, 8). In other words, the formation of pathognomonic autoantibody was related to symptoms. Such a development has never been detected, with research on CSF autoantibodies from DSDD yielding negative results (4). Although this is an experience of single case, we speculate that the establishment of NMDARE may not be incidental, but may be linked to the neuroinflammatory aspects of DSDD. Catatonia in DS had an extremely high recurrence rate and required long-term medication and/or several courses of electroconvulsive therapy to maintain recovery, when compared to catatonia caused by other psychiatric and medical conditions (16). Persistent systemic and intracerebral inflammation has been seen in DS patients (17, 18), which may be causally linked to the development of DSDD, as well as to such a protracted psychiatric illness (4). The neuroinflammation innate to DS could have contributed to the formation of anti-NMDAR antibodies in the current patient. Having seen the sequential emergence of typical DSDD and definite NMDARE with ambiguous temporal boundary, we could consider this case to be post-DSDD NMDARE, similar to the concept of post-HSV encephalitis NMDARE (14). The current example may elicit repeated investigation of CSF antibodies in selected cases with prolonged and/or unique clinical courses.

Since different sets of CSF cytokines/chemokines have been examined in each study, the comparison of this aspect between NMDARE and DSDD was quite difficult (Supplementary Table S1). However, the elevation of some inflammatory molecules at the initial phase with nonspecific complaint, 1 year before catatonic regression began, supports their roles in the pathogenesis of DSDD. Overdosage of certain gene alleles on the 21 chromosomes may be involved in the inherent mechanisms for neuroinflammation in DS. The autoimmune regulator (*AIRE*) gene product alters the phenotypic of lymphocytes in the thymus, perhaps leading to the development of anti-TPO autoantibody. The positive results of the anti-TPO antibody in the current case, as well as the higher prevalence of this antibody in DSDD, support the hypothesis of autoimmunity in the pathogenesis of DSDD (3, 4). Another candidate is the amyloid precursor protein (*APP*) gene, which is thought to develop the amyloid plaques with the resultant provocation of inflammation in DS brains. Amyloid plaques in DS can be detected on neuropathology as early as 8–9 years of age when there are no signs of dementia. From this perspective, it is intriguing that IL-5, IL-15, PDGFbb, and VEGF were found to be raised in the CSF of the current patient, all of which are upregulated in AD brains, but not in NMDARE in general (19–22). These findings support the concept that pre-AD disease contributes to the evolution of DSDD (3). Additionally, the biphasic elevation of certain chemokines characterizes the post-HSV encephalitis NMDARE (23, 24). This trend could also be observed in the values of IP-10/CXCL10 and MCP-1/CCL2, though not significantly increased compared to the reference ranges (Figure 3A). This last issue necessitates investigations on further DSDD subjects.

In conclusion, the clinical course of the current patient was thought to denote a transition from DSDD to NMDARE. Positive results of anti-NMDA receptor antibodies on the CBA coincided with this transition. Cytokine profiles in the CSF implied a precedent Alzheimer's pathology as a part of causative factors for DSDD. Although the exact mechanism of transition remains uncertain, repeated CSF examination for autoantibodies may be necessary in selected cases of DSDD with protracted and recurrent clinical course and/or with unusual neurological phenotype for DSDD.

## Data availability statement

The original contributions presented in the study are included in the article/Supplementary material, further inquiries can be directed to the corresponding author.

## Ethics statement

Ethical review and approval was not required for the study on human participants in accordance with the local legislation and institutional requirements. Written informed consent was obtained from the participant/patient(s) for the publication of this case report.

## Author contributions

YM, MS, FT, HM, MO, YO, AY, and YW were in charge of the clinical management of the subject and decisions concerning the treatment. YT examined the anti-NMDA receptor antibody assays and measured the cerebrospinal cytokines, chemokines, and other molecules. YM and YS wrote the draft. MS, YW, ST, and YT critically reviewed the manuscript. All authors have read and approved the final manuscript.
