# Supplementary material for: Case report: Evolution of catatonic mutism and psychotic symptoms in an adolescent with Down syndrome: transition from Down syndrome disintegrative disorder to anti-N-methyl-D-aspartate receptor encephalitis
Source: Front Neurol. 2023 Jun 9;14:1200541. doi: 10.3389/fneur.2023.1200541 (PMC10288866; doi:10.3389/fneur.2023.1200541)
Supplement: Supplementary file 2 [file Table_2.DOCX]

Table S2. Diagnostic criteria for childhood autoimmune encephalitis, NMDA receptor encephalitis, autoimmune psychosis, and catatonia.

**Diagnostic criteria for childhood autoimmune encephalitis**

[Cellucci T, Van Mater H, Graus F, Muscal E, Gallentine W, Klein-Gitelman MS, Benseler SM, Frankovich J, Gorman MP, Van Haren K, Dalmau J, Dale RC. Clinical approach to the diagnosis of autoimmune encephalitis in the pediatric patient. Neurol Neuroimmunol Neuroinflamm (2020) 7:e663. doi: 10.1212/NXI.0000000000000663. Table e-5]

**Possible childhood autoimmune encephalitis**

1. Acute or subacute onset of neurologic and/or psychiatric symptoms over ≤ 3 months in a previously healthy child

≥ 2 of the following clinical features:

• Altered mental status/level of consciousness, or EEG with slowing or epileptiform activity

(may be focal or generalized)

• Focal neurologic deficits

• Cognitive difficulties*

• Acute developmental regression

• Movement disorder (excluding tics)

• Psychiatric symptoms

• Seizures not explained by a previously known seizure disorder or other condition

Paraclinical testing may not be available

1. Serology Not available
2. Reasonable exclusion of alternative causes, including infectious

**Probable antibody-negative childhood autoimmune encephalitis**

1. Acute or subacute onset of neurologic and/or psychiatric symptoms over ≤ 3 months in a previously healthy child.

≥ 2 of the following:

• Altered mental status/level of consciousness, or EEG with slowing or epileptiform activity (may be focal or generalized)

• Focal neurologic deficits

• Cognitive difficulties*

• Acute developmental regression

• Movement disorder (excluding tics)

• Psychiatric symptoms

• Seizures not explained by a previously known seizure disorder or other condition

≥ 1 of the following:

• Presence of inflammatory changes in CSF (CSF leukocytosis > 5 cells/mm3, and/or CSF oligoclonal banding)

• MRI features suggestive of encephalitis

• Brain biopsy showing inflammatory infiltrates and excluding other disorders

1. Absence of autoantibodies associated with autoimmune encephalitis identified in serum and/or CSF
2. Reasonable exclusion of alternative causes, including infectious

**Definite childhood autoimmune encephalitis**

1. Acute or subacute onset of neurologic and/or psychiatric symptoms over ≤ 3 months in a previously healthy child

≥ 2 of the following:

- Altered mental status/level of consciousness, or EEG with slowing or epileptiform activity (may be focal or generalized)
- Focal neurologic deficits
- Cognitive difficulties*
- Acute developmental regression
- Movement disorder (excluding tics)
- Psychiatric symptoms
- Seizures not explained by a previously known seizure disorder or other condition

≥ 1** of the following:

- Presence of inflammatory changes in CSF (CSF leukocytosis > 5 cells/mm^3^, and/or CSF oligoclonal banding)
- MRI features suggestive of encephalitis
- Brain biopsy showing inflammatory infiltrates and excluding other disorders

1. Presence in the serum and/or CSF of well-characterized autoantibodies associated with autoimmune encephalitis
2. Reasonable exclusion of alternative causes, including infectious

* Severe cognitive dysfunction that is not attributable to a traditional psychiatric syndrome as documented by a qualified clinician (e.g. neurologist, psychiatrist, neuropsychologist), or a significant drop in IQ (>20 points)

** When CSF antibodies against N-methyl-D-aspartate receptor (NMDAR), gamma-aminobutyric acid A (GABA-A), glutamic acid decarboxylase 65 (GAD65) are present, further paraclinical markers of neuroinflammation are not required to diagnose definite AE.

**Diagnostic criteria for anti-NMDA receptor encephalitis**

[Graus F, Titulaer MJ, Balu R, Benseler S, Bien CG, Cellucci T, Cortese I, Dale RC, Gelfand JM, Geschwind M, Glaser CA, Honnorat J, Höftberger R, Iizuka T, Irani SR, Lancaster E, Leypoldt F, Prüss H, Rae-Grant A, Reindl M, Rosenfeld MR, Rostásy K, Saiz A, Venkatesan A, Vincent A, Wandinger KP, Waters P, Dalmau J. A clinical approach to diagnosis of autoimmune encephalitis. Lancet Neurol (2016) 15:391-404. doi: 10.1016/S1474-4422(15)00401-9.]

**Probable anti-NMDA receptor encephalitis**

Diagnosis can be made when all three of the following criteria have been met:

1. Rapid onset (less than 3 months) of at least four of the six following major groups of symptoms:

・Abnormal (psychiatric) behaviour or cognitive dysfunction

・Speech dysfunction (pressured speech, verbal reduction, mutism)

・Seizures

・Movement disorder, dyskinesias, or rigidity/abnormal postures

・Decreased level of consciousness

・Autonomic dysfunction or central hypoventilation

1. At least one of the following laboratory study results:

Abnormal EEG (focal or diffuse slow or disorganised activity, epileptic activity, or extreme delta brush)

CSF with pleocytosis or oligoclonal bands

1. Reasonable exclusion of other disorders

Diagnosis can also be made in the presence of three of the above groups of symptoms accompanied by a systemic teratoma

**Definite anti-NMDA receptor encephalitis**

Diagnosis can be made in the presence of one or more of the six major groups of symptoms and IgG anti-GluN1 antibodies, after reasonable exclusion of other disorders

**Diagnostic criteria for autoimmune psychosis**

[Pollak TA, Lennox BR, Müller S, Benros ME, Prüss H, Tebartz van Elst L, Klein H, Steiner J, Frodl T, Bogerts B, Tian L, Groc L, Hasan A, Baune BT, Endres D, Haroon E, Yolken R, Benedetti F, Halaris A, Meyer JH, Stassen H, Leboyer M, Fuchs D, Otto M, Brown DA, Vincent A, Najjar S, Bechter K. Autoimmune psychosis: an international consensus on an approach to the diagnosis and management of psychosis of suspected autoimmune origin. Lancet Psychiatry (2020) 7:93-108. doi: 10.1016/S2215-0366(19)30290-1.]

**Possible autoimmune psychosis:**

The patient must have current psychotic symptoms of abrupt onset (rapid progression of <3 months) with at least one of the following:

• Currently or recently diagnosed with a tumour

• Movement disorder (catatonia or dyskinesia)

• Adverse response to antipsychotics, raising suspicion of neuroleptic malignant

syndrome (rigidity, hyperthermia, or raised creatine kinase)

• Severe or disproportionate cognitive dysfunction

• A decreased level of consciousness

• The occurrence of seizures that are not explained by a previously known seizure disorder

• A clinically significant autonomic dysfunction (abnormal or unexpectedly fluctuant

blood pressure, temperature, or heart rate)

If a patient has possible autoimmune psychosis, they should be investigated, including electroencephalography, MRI, serum autoantibodies, and cerebrospinal fluid (CSF) analysis (including CSF autoantibodies). The results should lead to a diagnosis of non-autoimmune psychosis or probable/definite autoimmune psychosis.

**Probable autoimmune psychosis:**

The patient must have current psychotic symptoms of abrupt onset (rapid progression of <3 months) with at least one of the seven clinical criteria listed above for possible autoimmune psychosis and at least one of the following:

• CSF pleocytosis of >5 white blood cells per μL

• Bilateral brain abnormalities on T2-weighted fluid-attenuated inversion recovery MRI

highly restricted to the medial temporal lobes

Or two of the following:

• Electroencephalogram encephalopathic changes (ie, spikes, spike-wave activity, or rhythmic

slowing [intermittent rhythmic delta or theta activity] focal changes, or extreme delta brush

• CSF oligoclonal bands or increased IgG index

• The presence of a serum anti-neuronal antibody detected by cell-based assay

After exclusion of alternative diagnoses.

**Definite autoimmune psychosis:**

The patient must meet the criteria for probable autoimmune psychosis with IgG class anti-neuronal antibodies in CSF.

Note that these criteria do not exclude a diagnosis being made in a patient with an acute

onset (<3 months) of psychosis, even if that patient has had a previous psychotic, other

psychiatric, or encephalopathic episode that resolved

**Diagnostic criteria for catatonia in DSM-5**

[American Psychiatric Association. Diagnostic and Statistical Manual of Mental Disorders (Fifth ed.). Arlington, VA: American Psychiatric Publishing; 2013. [doi: 10.1176/appi.books.9780890425596](https://doi.org/10.1176/appi.books.9780890425596)]

Catatonia is defined by the presence of three or more of the following:

1. Catalepsy (i.e., passive induction of postures held against the gravity)

2. Waxy flexibility (i.e., slight and even resistance to repositioning by the examiner)

3. Stupor (no psychomotor activity, no reactivity to the environment)

4. Agitation, not influenced by external stimuli

5. Mutism (i.e., no or minimal verbal response- not applicable in case of established aphasia)

6. Negativism (i.e., opposing or not responding to external stimuli or instructions)

7. Posturing (i.e., spontaneous and active maintenance of posture against gravity)

8. Mannerism (i.e., odd caricatures of ordinary actions)

9. Stereotypies (i.e., repetitive, frequent, non-goal directed movements)

10. Grimacing

11. Echolalia (i.e., repeating the words spoken by the examiner)

12. Echopraxia (i.e., mimicking of movements made by the examiner

**Bush-Francis Catatonia Rating Scale**

[Bush G, Fink M, Petrides G, Dowling F, Francis A. Catatonia. I. Rating scale and standardized examination. Acta Psychiatr Scand (1996) 93:129-36. doi: 10.1111/j.1600-0447.1996.tb09814.x.]

- Use the presence or absence of items 1-14 for screening.
- Use the 0-3 scale for items 1-23 to rate severity.

1. Excitement: Extreme hyperactivity, constant motor unrest which is apparently non-purposeful. Not to be attributed to akathisia or goal directed agitation.

0 =Absent. 1 =Excessive motion, intermittent. 2 =Constant motion, hyperkinetic without rest periods. 3 = Full-blown catatonic excitement, endless frenzied motor activity.

1. Immobility/stupor: Extreme hypoactivity, immobile, minimally responsive to stimuli.

0=Absent. 1 =Sits abnormally still, may interact briefly. 2 =Virtually no interaction with

external world. 3 =Stuporous, non-reactive to painful stimuli.

1. Mutism: Verbally unresponsive or minimally responsive.

0 =Absent. 1 =Verbally unresponsive to majority of questions; incomprehensible whisper.

2 = Speaks less than 20 word/min. 3 =No speech.

1. Staring: Fixed gaze, little or no visual scanning of environment, decreased blinking.

0 =Absent. 1 =Poor eye contact, repeatedly gazes less than 20 s between shifting of attention; decreased blinking. 2 =Gaze held longer than 20 s, occasionally shifts attention. 3 =Fixed gaze, non-reactive.

1. Posturing/catalepsy: Spontaneous maintenance of posture(s), including mundane (e.g. sitting or standing for long periods without reacting).

0 =Absent. 1 =Less than 1 min. 2=Greater than one minute, less than 15 min. 3 =Bizarre posture, or mundane maintained more than 15 min.

1. Grimacing: Maintenance of odd facial expressions.

0 =Absent. 1 =Less than 10 s. 2 =Less than 1 min. 3 =Bizarre expression(s) or maintained more than 1 min.

1. Echopraxia/echolalia: Mimicking of examiner’s movements/speech.

0 =Absent. 1 =Occasional. 2 =Frequent. 3 =Constant.

1. Stereotypy: Repetitive, non-goal-directed motor activity (e.g. finger-play; repeatedly touching, patting or rubbing self); abnormality not inherent in act but in its frequency.

0 =Absent. 1 =Occasional. 2 =Frequent. 3 =Constant.

1. Mannerisms: Odd, purposeful movements (hopping or walking tiptoe, saluting passers-by or exaggerated caricatures of mundane movements); abnormality inherent in act itself.

0 =Absent. 1 =Occasional. 2 =Frequent. 3 =Constant.

1. Verbigeration: Repetition of phrases or sentences (like a scratched record).

0 =Absent. 1 =Occasional. 2 =Frequent, difficult to interrupt. 3 =Constant.

1. Rigidity: Maintenance of a rigid position despite efforts to be moved, exclude if cog-wheeling or tremor present.

0 = Absent. 1 = Mild resistance. 2 = Moderate. 3 = Severe, cannot be repostured.

1. Negativism: Apparently motiveless resistance to instructions or attempts to

move/examine patient. Contrary behavior, does exact opposite of instruction.

0= Absent. 1= Mild resistance and/or occasionally contrary. 2 = Moderate resistance and/or frequently contrary. 3 =Severe resistance and/or continually contrary.

1. Waxy flexibility: During reposturing of patient, patient offers initial resistance before allowing

himself to be repositioned, similar to that of a bending candle.

0 = Absent. 3 = Present.

1. Withdrawal: Refusal to eat, drink and/or make eye contact.

0 = Absent. 1 = Minimal PO intake/interaction for less than 1 day. 2 = Minimal PO intake/interaction for more than 1 day. 3 = No PO intake/interaction for 1 day or more.

1. Impulsivity: Patient suddenly engages in inappropriate behavior (e.g. runs down hallway,

starts screaming or takes off clothes) without provocation. Afterwards can give no, or only a facile explanation.

0 =Absent. 1 = Occasional. 2 = Frequent. 3 = Constant or not redirectable.

1. Automatic obedience: Exaggerated cooperation with examiner’s request or spontaneous

continuation of movement requested.

0 = Absent. 1 = Occasional. 2 = Frequent. 3 = Constant.

1. Mitgehen: “Anglepoise lamp” arm raising in response to light pressure of finger, despite

instructions to the contrary.

0 =Absent. 3 =Present.

1. Gegenhalten: Resistance to passive movement which is proportional to strength of the

stimulus, appears automatic rather than wilful. 0 =Absent. 3 =Present.

1. Ambitendency: Patient appears motorically “stuck” in indecisive, hesitant movement.

0 =Absent. 3 =Present.

1. Grasp reflex: Per neurological exam. 0 =Absent. 3 =Present.
2. Perseveration: Repeatedly returns to same topic or persists with movement.

0 =Absent. 3 = Present.

1. Combativeness: Usually in an undirected manner, with no, or only a facile explanation

afterwards.

0 = Absent. 1 = Occasionally strikes out, low potential for injury. 2 = Frequently strikes out, moderate potential for injury. 3 = Serious danger to others.

1. Autonomic abnormality: Circle: temperature, BP, pulse, respiratory rate, diaphoresis.

0 = Absent. 1 = Abnormality of one parameter [exclude pre-existing hypertension]. 2 = Abnormality of two parameters. 3 = Abnormality of three or more parameters.
